# Coping and Resilience Among Endurance Athletes During the COVID-19 Pandemic

**DOI:** 10.3389/fpsyg.2022.811499

**Published:** 2022-05-19

**Authors:** Brian Harman, Grégory Dessart, Liene Puke, Roberta Antonini Philippe

**Affiliations:** ^1^Nottingham Business School, Nottingham Trent University, Nottingham, United Kingdom; ^2^Institute for Social Sciences of Religions, FTSR, University of Lausanne, Lausanne, Switzerland; ^3^Service of Adult Psychiatry North-West, Department of Psychiatry, Lausanne University Hospital (CHUV), Lausanne, Switzerland; ^4^Institute of Sport Sciences, SSP, University of Lausanne, Lausanne, Switzerland

**Keywords:** COVID-19, pandemic, coping, resilience, athletes, endurance

## Abstract

COVID-19 lockdowns constrained the training opportunities of athletes resulting in physical and mental hardship. In this study, athletes involved in the outdoor endurance sports of running, cycling or swimming were recruited through Facebook groups and using online mailing lists. The final sample (*n* = 3,551) consisted of 576 female respondents (16.2%), and 2,975 male respondents (83.8%). The mean age of participants was 44.13  years (min = 16, max = 83, and *SD* = 9.84). An online survey was designed to measure variables relevant to athletes’ mental health; resilience and emotion regulation strategies; mobility restrictions; training routines; personal involvement in endurance sports; age; gender; and country of residence. Overall, the results of our study indicate that during lockdown, decreases in training volume, lower lockdown-specific resilience, and holding more negative perceptions about lockdown mobility restrictions (perceived strictness) all contributed to perceived barriers to training. In the analysis, athletes’ relative observance of mobility restrictions was controlled for. Athletes exhibiting high personal commitment to their sports displayed: greater lockdown resilience, a greater use of adaptive coping strategies, and lower levels of perceived barriers to training.

## Introduction

“The coronavirus COVID-19 pandemic is the defining global health crisis of our time and the greatest challenge we have faced since World War II” (United Nations Development Programme, 2021). As of February 20, 2022, over 424 million people have contracted COVID-19 resulting in over 5.8 million deaths in 192 countries (John Hopkins University, 2022). Alarmingly, new research suggests that deaths linked to the pandemic may actually be three times higher than official estimates suggest (Wang et al., 2022). However, less contentious are the psychological costs of a pandemic. A review of 24 studies suggests the psychological effects of a pandemic are wide ranging and often longstanding (Brooks et al., 2020). Lockdown measures across the globe radically transformed the everyday lives of hundreds of millions of people, creating psychological stress and hardship at an unprecedented scale (Abbot, 2021).

Confinement and isolation are detrimental to psychological well-being (Chen and Feeley, 2014). Past research has demonstrated that pandemics induce a variety of negative emotional states (e.g., Mihashi et al., 2009; Yoon et al., 2016). The stress and social isolation that characterise a lockdown can induce feelings of irritability and guilt (Lee et al., 2005), anger (Marjanovic et al., 2007), and stress (DiGiovanni et al., 2004) and can be detrimental to mental and cardiovascular health (Haslam et al., 2018). For example, in Australia, reports of poor mental health more than doubled among the general population during lockdown (Fisher et al., 2020). Similar, negative effects on public mental health were observed in Japan (Shigemura et al., 2020), Italy (Pagnini et al., 2020; Rossi et al., 2020), and in the United Kingdom (YouGov, 2020). In the United Kingdom, the number of people reporting feeling “happy” halved (from 50% to 26%) during the first month (March 2020) of lockdown (YouGov, 2020). Over the same period, the percentage of people who reported feeling scared tripled (from 11% to 34%).

COVID-19 lockdowns have led to reduced wellbeing (Lades et al., 2020; Yang and Ma, 2020), increased stress (Di Fronso et al., 2020), and mental health issues (Brooks et al., 2020; Pfefferbaum and North, 2020; Rajkumar, 2020; Rossi et al., 2020). Indeed, recent studies have catalogued a plethora of negative psychological outcomes resulting from pandemic related confinement (Chtourou et al., 2020; de Oliveira Neto et al., 2020; Duan et al., 2020; Qiu et al., 2020; Rajkumar, 2020; Shigemura et al., 2020; Varshney et al., 2020; Wang et al., 2020). This is unsurprising since past research on the psychological impact of pandemics has found similar effects (e.g., Hawryluck et al., 2004).

### Stress

Stress can be defined as “the quality of experience, produced through a person-environment transaction that through either over-arousal or under-arousal results in psychological or physiological distress” (Aldwin, 2007 p. 24). Selye (1956) described “stress” as anything that can significantly affect an individual’s “homeostasis” (see Cannon, 1929). Prolonged stress may promote disease (Schneiderman et al., 2005) and can increase an individual’s vulnerability to mental and physical illness (Caplan, 1981). In recent times, anxiety and depression symptoms (16%–28%) and self-reported stress (8%) are among the most commonly reported psychological reactions to lockdown (Rajkumar, 2020). Data from China indicate that stress (32.2%), anxiety (36.3%) and depression symptoms (30.3%) were commonplace during the early stages of the lockdown (Wang et al., 2020). In addition, disrupted sleeping patterns and sleep deprivation are symptomatic of stress (Lee et al., 2005) and have been observed during the COVID-19 lockdown (Stanton et al., 2020). Regardless of an individual’s age, it appears that emotional disturbance, anxiety, depression, and loneliness are found to characterise the lockdown experience of many (Andreato et al., 2020; Chtourou et al., 2020; Frank et al., 2020; Mehrsafar et al., 2020; Schinke et al., 2020; Wang et al., 2020). Indeed, Stanton et al. (2020) found that individuals reported increased alcohol consumption (26.6%) and smoking (6.9%) to help counteract lockdown stress. Lockdown conditions also appear to increase the risk of over-consumption and poor diet choice (Scarmozzino and Visioli, 2020).

### Physical Activity During Lockdown

A cross cultural study of health behaviours across eight countries (*n* = 1,131) revealed that decreases in reported physical activity (PA) during lockdown were associated with lower mental and physical health (Ruiz et al., 2021). Similarly, in Australia, pandemic related stress had a detrimental effect on PA levels (48.9%) and sleeping patterns (40.7%). However, respondents reporting increased PA levels also reported significantly higher volumes of sleep and lower weight gain. It appears PA counteracts many of the adverse effects of lockdown.

The World Health Organisation (WHO) recommends at least 150–300 mins of moderate-intensity PA or at least 75–150 mins of high intensity PA per week (WHO, 2021). However, during the COVID-19 pandemic, PA levels are found to vary widely across different populations and cohorts. While some studies reported an increase in PA during lockdown (Brand et al., 2020; Ding et al., 2020; Schnitzer et al., 2020; Smith et al., 2020), many other studies reported a decline in PA (Ammar et al., 2020; Castañeda-Babarro et al., 2020; Jiménez-Pavón et al., 2020; Lesser and Nienhuis, 2020; López-Bueno et al., 2020; Meyer et al., 2020; Mon-López et al., 2020; Qin et al., 2020; Sá Filho et al., 2020; Stanton et al., 2020; Xiang et al., 2020).

Research suggests that during the early stages of lockdown, many people responded in an adaptive manner and enthusiastically embraced exercise as a means of coping with stress. For example, during the first United Kingdom lockdown, 75% of respondents reported undertaking sufficient exercise to meet the recommended WHO PA guidelines (Smith et al., 2020). However, it is less clear if this initial enthusiasm for exercise was maintained during the prolonged months of lockdown. It appears that for many people, the enforcement of lockdown conditions coincided with a marked decrease in PA. Indeed, in the United States, there was a 48% reduction in PA during lockdown (COVID-19 Pulse, 2020). Correspondingly, data from Fitbit (2020) revealed a widespread decline in walking (steps) during the last week of March 2020 (when lockdowns were first introduced across Europe). When compared to data from the same period the previous year, analysis revealed a significant decline in walking—as much as a 38% decline in Spain. The following month there was a 12% reduction in total worldwide steps during April 2020 as people transitioned to indoor exercise (Garmin, 2020).

Reduced PA during lockdown increases sedentary behaviours and promotes unhealthy eating habits (Robins et al., 2018; Werneck et al., 2019; Ammar et al., 2020). Reduced PA also negatively affects mental and physical health (Cheval et al., 2021). Castañeda-Babarro et al. (2020) found that the greatest declines in PA were observed among males and young adults. This is interesting since research suggests that PA is more commonplace among younger, educated adults and less common among those who are older, less educated (Wennman et al., 2019) and who have lower incomes (Smith et al., 2020).

In Norway, research revealed that 24% of amateur and recreational athletes reported an increase in motivation to train while 41% of athletes reported no change in motivation and 35% reported they were less motivated (Rubio et al., 2020). Although the authors assessed motivation to train (rather than physical activity), they noted that their data align with the findings of larger studies. For example, Brand et al. (2020) found that 32% of respondents reported an increase in exercise (while 44% reported no change in training and 24% reported a decrease in activity). In contrast, a recent study by Mutz and Gerke (2021) found that only 9% of participants reported an increase in PA. Indeed, among this German sample, 49% of respondents reported a decrease in reported PA during lockdown. This lack of motivation to engage in PA is a cause for concern and may be linked to the lack of positive experiences with exercise in the past. Taken together, these studies suggest that between a quarter and a half of the population reduced their PA levels during lockdown.

Brand et al. (2020) revealed that athletes who reported higher volumes of training prior to lockdown were more likely to report higher levels of training motivation during lockdown. In line with past research (see Gerber et al., 2018), this finding supports the contention that regular exercisers benefit from an ability to access motivational resources despite situational stressors. Nevertheless, some studies did find that less active people became more active during lockdown (Brand et al., 2020; Schnitzer et al., 2020) and active people retained or improved upon pre-pandemic exercise levels (Brand et al., 2020). However, data from Canada suggest a more nuanced trend whereby lockdown conditions serve to reinforce pre-existing exercise tendencies (Lesser and Nienhuis, 2020). This finding suggests that while active people became more active, inactive people became more sedentary. Specifically, respondents who reported themselves as “active” were twice as likely to become more active (40%) than less active during the lockdown. The opposite trend was observed among respondents who reported themselves as inactive. Inactive individuals reported they were more likely to become less active (40%) than more active (33%). In short, the research by Lesser and Nienhuis (2020) suggests that lockdown conditions tend to reinforce pre-existing behaviours patterns by activating or inhibiting certain response tendencies. Understanding how and why and to what extent athletes exercise during lockdown will help us to gain a greater understanding of how they cope with barriers to training.

### Coping

Coping is the “constantly changing cognitive and behavioural efforts to manage specific external and/or internal demands that are appraised as taxing or exceeding the resources of the person” (Lazarus and Folkman, 1984, p. 141). A classical way of categorising coping has been to divide them into problem-solving strategies and emotion-focused strategies (Lazarus and Folkman, 1984). More recent research suggests that there are three broad approaches to coping with stress; (a) task-oriented coping, (b) distraction-oriented coping, and (c) disengagement-oriented strategies (Compas et al., 2001). Other researchers have conceptualised coping strategies in terms of task focused strategies, behaviour focused strategies, emotion control strategies, or thought control strategies (Gould et al., 1993). While some research suggests that coping strategies might be viewed as effective or ineffective (Nicholls et al., 2005), other researchers assert that the effectiveness of any given coping strategy may fluctuate and be dependent upon the situational demands present (Lazarus, 1999). This perspective on the malleable nature of coping strategies suggests that an athlete’s coping strategy is contextually bound. A worldwide pandemic of the current scale provides an unprecedented context for exploring how athletes cope under these unique situational pressures.

Effective coping strategies are important in attenuating the negative effects of the pandemic on subjective well-being (Ye et al., 2020; Rettie and Daniels, 2021). A host of factors have been found to affect athletes’ coping strategies (Nicholls, 2016). For example, income (Belot et al., 2020; Pieh et al., 2020), socio-cognitive factors (Constant et al., 2020), and age (Girdhar et al., 2020) can determine the coping strategies employed to deal with lockdown stress. It is worth noting that these factors which promote adaptive coping may be interlinked. For example, research suggests that older people are least affected by negative emotions and implement effective emotion regulation strategies (Ruiz et al., 2021). This enhanced coping ability might be due to their cognitive maturity or might be due to their greater financial resources that insulate them against the financial stresses of lockdown. To cope with lockdown challenges, athletes may use adaptive coping strategies (e.g., “planning”) or maladaptive coping strategies (e.g., catastrophising) to deal with the loss of a purposeful and structured training regime. Past research has demonstrated that the cognitive maturity of athletes is found to determine the coping style adopted (Louvet et al., 2009). Specifically, the researchers found that athletes’ conscientiousness was positively (negatively) linked to task orientated (disengagement-oriented) coping. Other research has explored how cognitive social maturity influences the coping styles of athletes (Nicholls et al., 2013). Interestingly, research also suggests that older people are more adaptable to the constraints imposed by the pandemic. A Dutch study (*n* = 1,679) found no effect of the pandemic on the mental health of adults aged over 65 years (Van Tilburg et al., 2021). This finding may be explained by past research which suggests that older adults typically employ more effective coping strategies (Aldao et al., 2010). Specifically, older adults tend to use adaptive coping strategies (problem solving, reappraisal, and acceptance) which lend themselves well to overcoming the difficulties of lockdown (Livingstone et al., 2020). In contrast to many older adults, young people experience especially high levels of anxiety during lockdown (Di Renzo et al., 2020; Rodríguez-Rey et al., 2020; Liu et al., 2021). It appears that young people are especially vulnerable to the deleterious effects of confinement and report stronger negative emotions and greater mental distress than older adults (Terry et al., 2020). This is hardly surprising since a meta-analysis suggests that younger adults are more susceptible to anxiety and depression than older adults (Baxter et al., 2013). The high stress reported by young people may be symptomatic of their tendency to suppress their emotions and to adopt maladaptive coping responses such as rumination (Aldao et al., 2010). The lack of social support (peers) during lockdown may also be a factor affecting exercise rates during lockdown. The detrimental effects of lockdown on young adults are particularly pernicious since they are more financially and psychologically vulnerable than older cohorts. While young adults are less susceptible to the physical effects of COVID-19, they have few financial resources to buffer the financial consequences of the pandemic (Belot et al., 2020; Pieh et al., 2020). In addition to age and cognitive maturity, gender may also influence the coping strategies of athletes during lockdown. Research indicates that the emotion regulation strategies adopted during lockdown can differ between genders (León-Zarceño et al., 2021).

Research suggests that women may experience more negative emotions during lockdown than their male counterparts (Alsalhe et al., 2020; Fu et al., 2020; López-Bueno et al., 2020; Mon-López et al., 2020; Pieh et al., 2020; Rossi et al., 2020; Xionga et al., 2020). This gender difference in coping might be explained by females’ tendency to employ communally oriented coping strategies. For example, León-Zarceño et al. (2021) found that women are more reliant on coping strategies that employ emotional support. Thus, female athletes may be particularly vulnerable during lockdown when social contact becomes unavailable or inaccessible. The same may be true of young adults who are highly reliant on social interaction and socialisation processes during adolescence and early adulthood. Regardless of age and gender, athletes who adopt maladaptive emotion regulation strategies are likely to perceive higher barriers to training than athletes who adopt adaptive emotion regulation strategies. Similarly, we suggest that athletes who exhibit low levels of resilience will perceive higher barriers to training than athletes who exhibit high levels of resilience.

### Resilience

Resilience can be considered as a set of individual features that allows an individual to cope and even flourish when faced with adverse life events. If adversity leads to a disruption of biological and psychological homeostasis then different levels of personal resilience may result in varying outcomes; e.g., growth, regained homeostasis, lower homeostasis, or a dysfunctional state (Connor and Davidson, 2003). Sports psychology literature investigating resilience among elite athletes has defined psychological resilience as “the role of mental processes and behaviour in promoting personal assets and protecting an individual from the potential negative effect of stressors” (Fletcher and Sarkar, 2012, p. 675). This definition extends previous conceptual work in a number of ways and proposes a holistic and systematic approach. Specifically, five main families of psychological factors protect the best athletes from the potential negative effects of stressors; positive personality, motivation, confidence, focus, and perceived social support (Sarkar and Fletcher, 2014; Fletcher, 2021). Notably, recent research advocates a biopsychosocial approach to researching resilience whereby mental health considerations are incorporated into research designs (Reardon et al., 2019).

A recent review of resilience among elite athletes suggests that resilience is an emergent process that often depends upon leveraging social resources (Gupta and McCarthy, 2021). Unsurprisingly, past research has found that social support provides fortitude in challenging times (see Fletcher and Sarkar, 2012; Morgan et al., 2013; Brown et al., 2015; Codonhato et al., 2018). Sadly, social support is likely to be compromised during lockdown when contact with friends and family is strictly curtailed. In addition, the esprit de corps and sense of belonging that team sports provide is also largely lacking during lockdown (Hall, 2011; Meggs et al., 2016). Indeed, the lack of motivational input from peers and professionals is a serious concern for athletes who are left to their own devices during lockdown (Machida et al., 2013; Chacón-Cuberos et al., 2019). Interestingly, a study of Spanish university athletes competing at regional level found that their subjective vitality and autonomous (intrinsic) goal motives decreased due to lockdown (Martínez-González et al., 2021). However, the athletes’ controlled (extrinsic) goal motives were not affected during lockdown. In short, the results suggest that resilience positively predicted changes in subjective vitality through its effect on autonomous goal motives. The present study aims to consider resilience within the context of lockdown by adopting the holistic approach to resilience proposed by sports psychology literature. In our study, we extend this conceptualisation by applying it to (mainly) non-elite, endurance athletes under lockdown conditions. Accordingly, we assert it is important to not only examine an athlete’s general resilience potential but also to evaluate their subjective appraisals of lockdown specific restrictions (i.e., mobility restrictions).

Within the context of sport, adversity often presents itself as obstacles to training and competition. However, within the context of lockdown, this concept must be extended to encompass factors that contribute to additional (lockdown) hardship, namely mobility restrictions. Interestingly, terms such as “coping” and “resilience” appear to be closely linked constructs that have been used interchangeably within academic literatures relating to sport science and psychiatry. Helpfully, Fletcher and Sarkar (2013, p. 16) summarise the theoretical distinction as follows; “resilience is characterised by its influence on one’s appraisal prior to emotional and coping responses and by its positive, protective impact, whereas coping is characterised by its response to a stressful encounter and by its varying effectiveness in resolving outstanding issues.” The present study employs the concept of resilience not as an *a priori* factor but rather as a disposition that allows individuals to regain equilibrium through the maintenance of a goal-directed approach that promotes flourishing in the face of adversity (Connor and Davidson, 2003). By considering emotional regulation strategies (i.e., coping strategies) in a more general sense, the present study aims to investigate how athletes deal with lockdown hardship induced by unprecedented and often repressive, COVID-19 mobility restrictions.

### Hypotheses

In the present study, we hypothesise that athletes’ psychological responses to lockdown will determine their perceived barriers to training. Accordingly, we defined “perceived barriers to training” as the athlete’s subjective appraisal of the detrimental effects of lockdown on their training plans and routines. In this study, we hypothesise that athletes who use maladaptive coping strategies (i.e., poor emotion regulation strategies) and athletes that exhibit low lockdown resilience will display higher levels of perceived barriers to training. In addition, we hypothesise that athletes’ perception and response to mobility restrictions will influence their level of perceived barriers to training, since endurance athletes are accustomed to unfettered access to nature. Such restrictions will also likely affect both the volume of training and the type of training that athletes can undertake. As such, these mobility restrictions may be perceived as a setback to training. A large body of research suggests that PA provides numerous physiological and psychological benefits to individuals. Conversely, it should follow that any disruption to PA activities is likely to decrease wellbeing, thereby increasing perceived barriers to training. In line with past findings, we also hypothesise that age, gender, and the athletic level of the athlete will also contribute to perceived barriers to training. We aim to test two main hypotheses based on the aforementioned assumptions:

*Perceived barriers to training will be associated with:*

*H1a:* lower lockdown-specific resilience (RISC);
*H1b:* greater use of maladaptive and lower use of adaptive coping strategies (CERQ);
*H1c:* greater decrease in training hours during lockdown [training volume contrast (TVC); TVR]; and
*H1d:* greater negative perceptions of mobility restrictions (mobility restrictions perceived strictness; MRPS).

*Higher athletic levels (personal commitment to endurance sports) will be associated with:*

*H2a:* greater resilience and coping strategies;
*H2b:* lower perceived barriers to training.

## Materials and Methods

### Participants

Ethical approval for this project was obtained from the Faculty of Business and Law at De Montfort University prior to starting the study. Athletes involved in the outdoor endurance sports of running, cycling, or swimming were recruited through Facebook groups and online mailing lists. A substantial number of trail running participants were recruited through the International Trail Running Association (ITRA) who sent their members weblinks to the French and English versions of the online survey. The data were collected over a 19 day period (24/04/20–11/05/20) and a total of 5,331 athletes participated in the study. Participation in the study was voluntary and no incentive was provided to respondents. During the data cleaning process, the following exclusion criteria were applied: the removal of (1) any records with missing data; (2) novice endurance athletes (who may not experience training disruption due to mobility restrictions); (3) respondents who were not engaged in the outdoor endurance sports of running, cycling, or swimming; (4) respondents who reported that their sports activities are not important to them; and (5) respondents who reported more than one country of residence (which made mobility restrictions difficult to account for). The final sample (*n* = 3,551) consisted of 576 female respondents (16.2%), and 2,975 male respondents (83.8%). The mean age of participants was 44.13  years (min = 16, max = 83, and *SD* = 9.84).

The current study aimed to recruit endurance athletes who were likely to experience barriers to their training due to lockdown conditions. Consequently, endurance athletes involved in outdoor sports, such as running, cycling, and swimming were targeted during the recruitment stage. In addition, participants involved in other outdoor endurance sports, such as Nordic/cross-country skiing, rowing, and long-distance walking were also included in the study. Most participants practiced multiple endurance sports but running was found to be the most popular endurance sport undertaken; running (99.21%), cycling (56.74%), swimming (20.33%), long distance walking (28.74%), Nordic skiing/cross-country skiing/ski-mountaineering (13.83%) and rowing (1.17%).

Most participants in our study resided in France: mainland France (72.82%), Corsica (0.08%), French Guiana (0.03%), French Polynesia (0.03%), Guadeloupe (0.03%), Martinique (0.14%), Mauritius (0.06%), Mayotte (0.06%), New Caledonia (0.08%), and Reunion (0.98%). In addition, endurance athletes from across European participated in the study: Andorra (0.03%%), Belgium (1.04%), Channel Islands (0.08%), Czech Republic (0.06%), Denmark (0.08%), Estonia (0.06%), Germany (0.11%), Ireland (1.77%), Italy (0.08%), Latvia (1.18%), Luxembourg (0.22%), Montenegro (0.03%), Norway (0.03%), Portugal (0.03%), Serbia (0.03%), Spain (0.17%), Sweden (0.17%), Switzerland (7.60%), The Netherlands (0.14%), and United Kingdom (11.60%). Endurance athletes from outside Europe also participated in the research; Australia (0.08%), Canada (0.08%), Costa Rica (0.03%), Hong Kong (0.03%), Mexico (0.03%), New Zealand (0.03%), Oman (0.03%), Russia (0.25%), South Africa (0.08%), Thailand (0.03%), Turkey (0.03%), United Arab Emirates (0.03%), United States (0.37%), and Vietnam (0.03%). In all, our sample consisted of 3,551 endurance athletes across 21 different countries. Athletes from France and the United Kingdom (areas with very strict mobility restrictions) represented the vast majority (84.42%) of participants in the study.

### Procedure and Methods

The current study took a series of variables into consideration: resilience and emotion regulation strategies; mobility restrictions; training routines; personal involvement in endurance sports; age; gender; and country of residence. Both an English and a French version of the measures were used in the corresponding versions of the online survey.

#### Resilience and Emotion Regulation Strategies

Athlete resilience was assessed using the Connor-Davidson Resilience Scale (RISC-25; see Connor and Davidson, 2003). Instructions were slightly modified to frame resilience within the context of the current pandemic; “your answers should reflect how you feel about your confinement during the current COVID-19 crisis.” Participant’s lockdown resilience was then measured by assessing participants’ agreement with the standard 25 statements (e.g., “I am able to adapt when changes occur.”). A five-point Likert scale was used, ranging from “not true at all” to “true nearly all the time.” Participants’ scores were totaled such that higher scores indicated higher levels of resilience. Resilience scores were then reversed for the regressions analysis to align with the same expected direction of the other predictors. The RISC-25 indicated high internal consistency (Cronbach’s *α* = 0.86).

Cognitive, emotion regulation strategies were assessed using the Cognitive Emotion Regulation Questionnaire (CERQ; Garnefski and Kraaij, 2007). Participants rated all 36 statements (e.g., “I think that other people go through much worse experiences.”), using a five-point Likert scale, ranging from “(almost) never” to “(almost) always.” The CERQ uses two subscales: (CERQ adaptive and CERQ maladaptive) to measure adaptive and maladaptive coping responses, respectively. Participant’s scores for both subscales were totaled and scores for the CERQ adaptive subscale were reversed for the regression analyses. Both the CERQ adaptive and CERQ maladaptive subscales demonstrated high internal consistency (Cronbach’s *α* = 0.89 and 0.83, respectively).

#### Training Routine

Participants were asked to report their average weekly hours of running, cycling, and swimming before the lockdown and during the lockdown. The scores for the number of training hours before and during the lockdown were then compared to calculate differences in training volume (TVC). Thus, a negative score indicates a decrease in a participant’s training volume during lockdown. Scores were reversed for the regression analyses, with higher positive scores indicating a higher decrease in training hours. Participants were also asked to provide information about the changes and obstacles to their training routine (open answers). The open answers were analysed using thematic analysis.

#### Mobility Restrictions

Participants were asked to evaluate: (1) how difficult they found it to comply with mobility restrictions in their area (mobility restrictions perceived strictness); (2) how strictly they had adhered to these restrictions (mobility restrictions observance); (3) how disruptive the lockdown had been to their physical training (mobility restrictions as detrimental to one’s training); and (4) how much their training routine had changed during lockdown (changing training routine during lockdown). For all four measures, a five-point Likert scale was used, ranging from “not at all” to “extremely.”

#### Age and Gender

All participants reported their age and gender to allow us to explore the possibility of age and gender effects in the data.

##### Personal Commitment to Endurance Sports

Participants were asked to categorise themselves into one of the following groups; a novice, an amateur, a competitive amateur, or a semi-professional/professional athlete. Participants who self-reported as a novice was removed from the sample since the impact of mobility restrictions were unlikely to affect their training regimes.

### Data Analyses

A multiple regression analysis was performed to determine which variables contributed to athlete’s perceived barriers to training during the COVID-19 lockdown. Spearman’s rho tests were conducted in order to assess the effect of athletic level on resilience, coping, and the degree of perceived barriers to training. A qualitative analysis based on open-ended questions was conducted to identify changes to training routines and the barriers to training. All tests were performed using SPSS (version 26).

## Results

The following categories of possible contributors to “perceived barriers to training” were considered: psychological factors (resilience and coping strategies of athletes), behavioural factors (changes to the training volume), and perceptual/behavioural responses to mobility restrictions.

### Descriptive Statistics for Focal Variables

Descriptive statistics of the sample are shown in Table 1.

**Table 1:** Descriptive statistics of the sample.

	Minimum	Maximum	Mean	*SD*	Variance
Weekly hours of exercise (running, cycling, or swimming) during lockdown	0.00	100.00	6.61	7.022	49.31
Weekly hours of exercise (running, cycling, or swimming) before lockdown	0.00	90.00	8.87	6.41	41.05
Contrast in weekly hours of exercise (running, cycling, and swimming) pre-lockdown *VS* lockdown	−56.00	94.00	−2.26	6.54	42.74
RISC total score	14.00	100.00	71.05	10.28	105.70
CERQ adaptive	20.00	100.00	67.65	11.88	141.18
CERQ maladaptive	16.00	76.00	32.14	8.15	66.47

### Effects of Mobility Restrictions on Perceived Barriers to Training

Shapiro–Wilk tests revealed that none of the predictor variables were normally distributed so nonparametric statistics were used to test H1. Spearman’s rho tests were used to identify significant relationships between athletes’ perceived barriers to training and the following variables: adaptive cognitive emotion regulation (CERQ adaptive), maladaptive cognitive emotion regulation (CERQ maladaptive), and resilience (RISC). While CERQ adaptive did not show any significant relationship, analysis revealed a positive significant relationship with CERQ maladaptive, *r_s_* = 0.04, *p* < 0.02, and a negative significant relationship with RISC, *r_s_* = −0.06, *p* < 0.001. This result suggests that low lockdown resilience and maladaptive coping responses contributed to perceived barriers to training. Further analysis was conducted to determine the role of these variables after accounting for other possible contributors. Thus, a multiple linear regression analysis was used to construct a model predicting how severe athletes perceived the barriers to training. Specifically, training volume contrast (TVC rev.), adaptive cognitive emotion regulation reversed score (CERQ adaptive rev.), maladaptive cognitive emotion regulation (CERQ maladaptive), resilience reversed score (RISC rev.), mobility restrictions perceived strictness (MRPS), and observance of mobility restrictions (OMR) were included in the regression model. Scores were reversed for the RISC in order to follow the same expected direction as other predictors. This analysis was conducted to determine if athlete’s perceptions of training setbacks resulting from mobility restrictions would depend upon athlete’s ability to cope (resilience, emotion regulation, and observance of mobility restrictions), their subjective evaluation of the situation (mobility restrictions perceived strictness), and the athlete’s objective impact of the mobility restrictions on their training volume (TVC). The results of the multiple regression analysis are reported in Table 2.

**Table 2:** Multiple regression predicting perceived barriers to training.

Predictors	Zero-order *r*	*β*	*t*	*b*
(Constant)		0.786^**^	5.643	
TVC rev.	0.248	0.028^**^	12.167	0.174
CERQ adaptive rev.	0.011	0.000	0.092	−0.002
CERQ maladaptive	0.040	0.001	0.275	0.004
RISC rev.	0.062	0.003^*^	1.989	0.032
MRPS	0.470	0.469^**^	32.062	0.460
OMR	0.171	0.250^**^	12.840	0.197

* *p* < 0.05;

** *p* < 0.001.

It was hypothesised that perceived barriers to training would be predicted by: lockdown-specific resilience *reversed score* (RISC rev; H1a), CERQ adaptive rev., maladaptive cognitive emotion regulation (CERQ maladaptive; H1b), training volume contrast *reversed score* (TVC rev; (H1c), and mobility restrictions perceived strictness (MRPS; H1d). OMR was used as a control variable, as this could have a direct impact on the way one experiences a lockdown situation. Individuals who failed to observe mobility restrictions would likely experience less stress than other athletes since they could continue their training as normal. To test these hypotheses, we conducted a multiple linear regression analysis. The regression model explained 29.4% of the variance of perceived barriers to training, *F*(6, 3,544) = 247.087, *p* < 0.001. Significant individual contributions of the predictors showed that TVC rev. (*p* < 0.001), RISC rev. (*p* < 0.05), MRPS (*p* < 0.001), and OMR (*p* < 0.001) predicted higher levels of perceived barriers to training. Mann–Whitney U tests were used in order to test the possible impact of gender on: RISC, MRPS, perceived barriers to training, and OMR. Only one significant difference based on gender was found for OMR, *p* < 0.025, suggesting that female athletes (mean rank = 1857.46) showed a greater observance of mobility restrictions than their male counterparts (mean rank = 1760.23). Therefore, we can conclude that gender only had a minor influence on the variables under investigation. Our finding suggests that the levels of perceived barriers to training were higher when athletes reported: (1) a greater decrease in training volume, (2) lower lockdown-specific resilience, and (3) feeling that mobility restrictions were particularly strict. Predictors remained significant when taking into account the relative observance of mobility restrictions (which also remained a significant contributor to perceived barriers to training).

### Athletic Level and Resilience Coping and Perceived Barriers to Training

Non-parametric statistics were used to test H2. Spearman’s rho tests were used to identify significant relationships between athletic level, on the one hand, and lockdown resilience (RISC), adaptive coping strategies (CERQ; H2a), and perceived barriers to training (H2b), on the other hand. Non-parametric statistics were used. Athletic level shared a positive significant relationship with CERQ adaptive, *r_s_* = 0.06, *p* < 0.001, and RISC, *r_s_* = 0.11, *p* < 0.001, and a negative significant relationship with perceived barriers to training, *r_s_* = −0.03, *p* = 0.48. Importantly, no difference could be observed in TVC based on athletic level. This finding suggests that among athletes who were already committed to endurance sports before the lockdown (those exhibiting higher athletic levels and therefore higher personal commitment to sports) are associated with: (1) greater lockdown resilience, (2) greater use of adaptive coping strategies, and (3) lower perceived barriers to training.

### Thematic Analysis of Qualitative Data

In response to open questions, participants provided information about the changes and obstacles to their training routine. Analysis of these qualitative data revealed two distinct themes; (1) Obstacles to training and (2) Changes in training. Taken together, these themes explore; (1) COVID related constraints to training and (2) the changes and adaptations employed to overcome these constraints. In the following sections, these themes and sub-themes are discussed to provide additional insights into the coping strategies of endurance athletes during COVID-19 lockdown conditions. We analysed 331 open ended answers that related specifically to the obstacles to training theme identified (female = 60, male = 271). Similarly, with regard to changes in athlete’s training, all 1,665 responses received from athletes were analysed (female = 275, male = 1,390). The response ratio roughly corresponds to the gender ratio in the overall sample.

A thematic inductive approach was employed to analyse the data, which involved different steps (Braun and Clarke, 2006; Braun et al., 2016). The answers were first read through to familiarise the researchers with their content. Analysis involved identifying and dividing the transcripts into meaning units: parts of text representing a single idea in relation to the research question (Robson, 2011). These meaning units were labelled and then reviewed across all of the transcripts to check for consistency across the dataset. Next, the labelled meaning units were grouped into categories and themes with other similar meaning units. No software was employed in any stage of the analysis; instead, all coding and grouping was conducted by hand. Steps were taken to establish and ensure the reliability of the analysis process and the emergent findings. Discussion among the research team reviewed the resulting themes following the analysis process (Smith and McGannon, 2018). When disagreements occurred, consensus was sought through discussion and cross-referencing with the interview transcripts. To ensure that the researchers’ interpretation did not go beyond what was actually said by participants’ quotes are provided throughout the results section so that readers may form their own judgements on their meaning.

#### Theme 1: Obstacles to Training

This first theme explores the various reasons which prevented athletes from training during the lockdown. The obstacles to training can be categorised into three sub-themes, which are: (a) intraindividual obstacles; (b) interindividual obstacles; and, (c) environmental obstacles.

##### Intraindividual Obstacles

Analysis of participants’ narratives suggest that intraindividual barriers to training to be categorised as moral, psychological, or physical in nature. Participants often reported not training out of respect for the sacrifice being made by health care staff (moral obstacles), due to fear of the virus (psychological obstacles) or due to an inability to train because of untreated injuries or sickness (physical obstacles).

“I was fearful of getting injured and taking up a space in hospital just because of my sport.” (A2707)

“Anxiety and anxiety took over.” (A11)

[I had a fear of]…“contracting COVID-19.” (A3537)

##### Interindividual Obstacles

Social pressure, work obligations and family commitments all created interindividual obstacles that were found to inhibit athletes’ ability to practice their sport activity. Transgressing COVID restrictions or deviating from new work practices contributed to the fear of social judgement thereby creating interindividual obstacles:

“Hatred against athletes and sports from other citizens, with the runner seen as a vector for the virus.” (A1859)

“Smart working takes much longer than normal, leaving very little time for other activities, including training.” (A622)

“Three kids at home who are not at school and their own sports, so my time has changed. [I am] given a guilt trip by my 12 year old if I want to go for a run.” (A2981)

##### Environmental Obstacles

This sub-theme captures the inability of participants to train due to the inaccessibility of the environment or facilities necessary to practice their sport.

“Removal of swimming from my sport, triathlon. …[I had] no more access to a pool or a lake.” (A26)

“I am unable to get to mountains to train and so hill training has taken a hit.” (A3415)

“Swimming pool closed; online equipment not available [to replicate swimming training].” (A3407)

#### Theme 2: Changes to Training

Participants identified four considerations that contributed to changes in their training regimes during lockdown. Specifically, athletes identified; (a) social interactions; (b) inability to enact typical training routines, (c) changes to the structure and type of training; and (d) a search for new forms of well-being.

##### Social Interactions

One of the main changes that participants consistently reported was a change in the number of social contacts during training. As participants moved from group training to solo training, their social circle contracted. However, new (online) social circles were often formed during lockdown as athletes searched for new training alternatives that complied with social distancing guidelines. Many participants took online sports classes such as yoga or strength training while others took part in app-based training or online challenges. Nevertheless, some participants implemented strategies designed to avoid all social contact during their training.

“[I now do] online circuit classes rather than driving to hilly trails.” (A2134)

“I normally train with groups so training on my own is difficult.” (A2925)

“I have to run at night around a golf course to avoid other people and more importantly, the police.” (A3047)

##### Inability to Enact Typical Training Routines

During lockdown, participants’ reported temporal and geographic constraints on their normal training routines. A lack of access to mountainous areas and time constraints on their training schedules resulted in monotonous training regimes within their local areas. The inability to access nature often resulted in the athletes being obliged to train at erratic times and in locations that did not afford the training intensity they had become accustomed to enjoying in the past.

“I was running in hamster mode within a mile of home.” (A1387)

“[My time schedule has changed, more rides and runs at other times of the day]….less sticking to a schedule.” (A2917)

“[…] My wife is unable to share in walking our dog. This means I need to go out for an hour training with our dog everyday with no rest days.” (A3451)

##### Changes to the Structure and Type of Training

As expected, participants reported widespread changes to the structure and type of training activity they performed during lockdown. Indeed, many participants reported that while they continued to perform the same sport training they were forced to modify the training load. While some were able to increase the volume, frequency, or intensity of their training, others saw their training load decrease significantly. Some participants were unable to practice their sports due to the closure of some facilities and therefore engaged in new sports and physical activities. Many participants started working out at home using home trainers, treadmills, and other devices that allowed them to exercise in different ways. Interestingly, several athletes reported that changes in their sporting goals had a direct impact on the way they trained. The changes in training goals were largely explained by the removal of races and competitive sporting events.

“I am training more frequently as I am working from home. No commute and more flexible working hours mean I am able to train when I want.” (A3539)

“No race-focused preparation—much more general, less structured running.” (A3544)

“My training is defined by the official competitions I participate in (12–15 per year). No competition in the medium term, so less consistency in my training.” (A936)

##### A Search for a New Form of Well-Being

Lockdown conditions prompted many athletes to reassess their physical and psychological well-being. Some athletes reported that the lockdown provided them with an unprecedented (but welcome) opportunity to rest their bodies. It also made the athletes more cognizant of their general health and the newfound threat associated with the COVID-19 virus.

“I stopped my training plan so that I only run for fun now.” (A2708)

“[I need] to carry necessary protective equipment (to cover my face).” (A2905)

“[…]…taking advantage of the confinement to rest the body and have it repair all its little hurts that we do not take the time to do during the season.” (A986)

## Discussion

Gupta and McCarthy (2021) assert that the resilience of elite athletes is frequently context dependent and often reliant upon resource availability. However, any individuals who experience resource scarcity can find it difficult to pursue goals associated with health and wellbeing (Mullainathan and Shafir, 2014). A global pandemic provides a unique context for examining resilience and coping strategies since many of the resources typically available to athletes become inaccessible. For endurance athletes living through lockdown, scarcity manifests itself as an inability to access both training resources and psychological support. Past research has demonstrated that communities can share psychological burdens by sharing resources, thereby fostering “community resilience” (Norris et al., 2011). However, during a lockdown situation, the ability to share resources is limited. In such challenging times, it is the lockdown resilience of the athlete and the coping strategies they employ that determine their perceived barriers to training.

Research suggests that the COVID-19 pandemic has created high levels of psychological distress in the general population. For example, one Australian study found that 33% of respondents (*n* = 1,062) “were at increased risk of experiencing some form of clinically diagnosable mood related disorder” (Terry et al., 2020, p. 6). Similarly, an Australian study by Fisher et al. (2020) found that 25% of respondents reported mild to moderate depression symptoms when lockdown was introduced. These figures are alarmingly high when compared to the normal incidents of mood disorders. A meta-analysis of 148 studies suggests that the global prevalence of mood disorders is 5.4% (Steel et al., 2014). Importantly, a meta-analysis by Schuch et al. (2019) has shown that PA protects individuals from high levels of anxiety. Whatsmore, recent studies provide evidence that PA helps to attenuate the negative psychological effects of lockdown (Ferreira-Júnior et al., 2020; Slimani et al., 2020; Teychenne et al., 2020), which are found to compound pre-existing problem behaviours such as gambling among elite athletes (Håkansson et al., 2020).

During lockdown, normal PA routines are severely disrupted. Nevertheless, individuals who maintain physical activity during lockdown are found to exhibit better physical and psychological well-being than those who do not engage in exercise (Clemente-Suárez et al., 2020; Slimani et al., 2020). Arguably, lockdown resilience and effective coping are especially important for endurance athletes since they have become accustomed to the benefits of regular exercise and may therefore be especially sensitive to decreases in PA. Researchers have observed “an almost linear dose–response relationship between exercise frequency and mood” (Brand et al., 2020 p. 7).

In the current study, we found that athletes reduced their training hours during lockdown. This reduction in training hours is indicative of elevated barriers to training. Importantly, we observed a significant relationship between reduced training volume and perceived barriers to training. This finding is in line with our expectations (H1c) and past research on PA. We also hypothesised that athletes’ perception of mobility restrictions would affect their perception of barriers to training. Again, in line with our expectations, athletes who perceived the mobility restrictions as being very strict were likely to perceive barriers to training as particularly onerous. Thus, H1d is supported. Analyses revealed that gender accounted for differences in mobility restriction observance. Female athletes were more likely to observe the imposed mobility restrictions than their male counterparts.

During lockdown, women are generally found to be more vulnerable to negative mood (Pieh et al., 2020; Rossi et al., 2020), more susceptible to negative emotional states (Alsalhe et al., 2020) and susceptible to greater emotional distress than their male counterparts (Terry et al., 2020). A similar pattern of results was reported in a recent Spanish study which also found that females experienced greater emotional disturbance (negative thoughts and negative mood) and poorer psychological well-being than males (León-Zarceño et al., 2021). In line with these generally observed trends, Ruffault et al. (2020) also found that female athletes exhibited higher rates of self-reported anxiety during the pandemic. We suggest that these results may be due to the fact that women are more sensitive to risk (Croson and Gneezy, 2009) and therefore may be more likely to experience greater negative emotions during lockdown. Lautenbach et al. (2020) recently investigated motivation and coping strategies and found that female athletes were more likely to experience demotivation than their male counterparts. However, both male and female athletes reported that they wished they could have received more support from their coaches. The COVID pandemic provided a climate of fear at an unprecedented scale but research suggests that “women are more inclined to seek emotional and instrumental support” when coping with lockdown stress (Szczypińska et al., 2021, p. 7). Interestingly, in the current study, female athletes did not differ from male athletes in terms of their lockdown resilience or coping strategies. However, it is worth noting that female athletes did report higher levels of adherence to mobility restrictions during the lockdown. This is hardly surprising since past research has demonstrated that women are more likely to engage in more prosocial behaviour than men (Abdullahi and Kumar, 2016). While women are more likely to observe mobility restrictions this does make them especially vulnerable during lockdown conditions. Research has shown that women tend to employ coping strategies that are based on communication with others (Szczypińska et al., 2021). When friends and family are deemed out of bounds, it appears that female athletes are less likely to flout mobility restrictions to access the social support they need. This unwillingness to break the rules might go some way to explaining the results of other studies, which suggest that women suffer greater psychological distress than men during lockdown. This might also be due to the coping strategies that female athletes use. Costa et al. (2020) found that while men are more likely to engage in “planning” and “blaming others,” female athletes are more likely to engage in “rumination” and “putting things into perspective.”

Recent research suggests that athlete’s competency (novice vs. elite) dictates the coping strategies employed during lockdown (Szczypińska et al., 2021). In the current study, we found that committed endurance athletes experienced lower levels of perceived barriers to training than less committed endurance athletes. Our analysis revealed that experienced endurance athletes exhibited high lockdown resilience which helped them overcome their perceived barriers to training. In addition, these experienced athletes also tended to use adaptive coping responses that also helped them during periods of confinement. This finding is in line with recent research that also used the CERQ measure to assess athletes’ coping responses during lockdown. Specifically, Costa et al. (2020) found that elite athletes responded in an adaptive manner by “accepting” the (lockdown) situation and by “planning” their response to it. In contrast, non-elite athletes were found to be susceptible to “self-blame.” This association between elite athletes and adaptive coping has also been observed in past research (Shirvani et al., 2015) and also more recently during the current pandemic (Leguizamo et al., 2021). It appears that elite athletes are more psychologically robust and adaptable than non-elite athletes. Leguizamo et al. (2021) found a negative association between adaptive coping strategies and negative emotional states. Across their sample of 310 high performance athletes in Europe, Asia, and America, the most effective coping strategies employed by athletes were “cognitive restructuring” and “emotional calming.” However, some of the athletes in the study were not endurance athletes (e.g., football, rugby, basketball, and martial arts) so they may not have been as reliant on accessing outdoor environments as the endurance athletes in the current study.

Contrary to expectation, athletes’ general coping strategies in the current study (i.e., athletes cognitive, emotion regulation as measured using the CERQ) did not influence athletes’ perceived barriers to training. While adaptive coping strategies (i.e., CERQ adaptive subscale) did not significantly influence perceptions of barriers to training, maladaptive coping strategies (i.e., CERQ maladaptive) were found to negatively affect perceived barriers to training. A regression model revealed a positive and significant relationship between low lockdown resilience and perceived setbacks to training. Specifically, athletes exhibiting low levels of lockdown resilience reported perceiving higher barriers to training. Thus, H1a is supported. However, this relationship did not remain statistically significant when accounting for other contributing variables in the regression model (mobility restrictions perceived strictness, observance of mobility restrictions, and contrast in training volume). Thus, H1b is not supported.

Recent research has found that gym going athletes needed time to adapt to the “new normal” (Rubio et al., 2020) and to home training during lockdown (Kaur et al., 2020). In the study, respondents reported that motivation and self-perception increased as they became accustomed to new training routines. However, for endurance athletes who frequent the outdoors, the transition to indoor training is likely to be more pronounced. The majority of the participants in the current study resided in countries where strict mobility restrictions were imposed. Athletes in France and in the United Kingdom (which constituted 84% of the sample) were forced to endure strict mobility restrictions that prevented them from accessing their natural training environments. Such mobility restrictions are less than ideal since exposure to nature is found to reduce stress (Triguero-Mas et al., 2017) and positively affect mood, self-esteem, and general happiness (Nguyen and Brymer, 2018). Indeed, exercising out of doors appears to have a number of advantages over indoor exercise. Individuals who exercise outdoors exhibit weaker negative states (tension, confusion, anger and depression) and exhibit stronger intentions to continue physical activity (Bowler et al., 2010). Outdoor exercise not only provides mood enhancing side effects but also boosts an individual’s cognitive performance (Berman et al., 2008). Individuals who frequent the outdoors also exhibit lower state and trait anxiety relative to their indoor inclined counterparts (Martyn and Brymer, 2016). The strict mobility restrictions imposed in France and the United Kingdom were in stark contrast to less populous countries (e.g., Finland) where less stringent restrictions were enforced. Interestingly, this policy of permitting unrestricted access to nature prompted a general increase in exercise and prompted 35% of participants (*n* = 601) to change the type of physical exercise routines. While gyms were closed, no restrictions were placed on outdoor exercise. Consequently, many individuals migrated their training regimes to outdoor environments (Ronkainen et al., 2021).

Open ended questions in our survey allowed us to gauge athletes’ perceptions and behaviours with regard to the imposed mobility restrictions. In the current study, athletes frequently reported that mobility restrictions compromised the quality of their training. Many reported that they became bored of their local area and missed the freedom and challenge of their normal training routes. While some respondents reported that the increased flexibility of remote working allowed them to train more frequently, many others reported that the lack of competitive events resulted in reduced training hours and less structured training plans. Nevertheless, some respondents reported that they liked the break in training and viewed the lockdown as an opportunity to rest, recuperate, and recover from past injuries. As expected, respondents reported that their social interactions with other athletes declined as they migrated from team-based training to solo training regimes and online classes. However, some athletes reported that the migration to web-based training did afford new opportunities for physical exercise. Nevertheless, these new training regimes presented little opportunity for meaningful social interaction with other athletes. Analysis of the qualitative data generated in response to open questions revealed two distinct themes among respondents; (1) obstacles to training and (2) changes to training. Three sub-themes were identified as obstacles to training; (a) intraindividual obstacles; (b) interindividual obstacles; and (c) environmental obstacles. The intraindividual obstacles encountered by athletes pertained to moral, psychological, and physical barriers that prevented them from maintaining their pre-lockdown training routines. Respondents reported that their sense of social solidity inhibited any willingness to flout COIVID-19 restrictions. In addition, legitimate fears with regard to contracting COVID-19 were also found to curb athletes’ training ambitions. Some respondents also reported that injuries and ill health prevented them from training. The absence of competitive sporting events was also found to reduce their motivation to train.

The second theme identified was changes to athletes training regimes during lockdown. Athletes identified four considerations that contributed to changes in their training regimes: (a) social interactions; (b) inability to enact typical training routines; (c) structure and type of training; and (d) a search for new forms of well-being. Past research has demonstrated that in times of adversity, endurance athletes form *ad hoc* groups to support their individual goals during races (Harman et al., 2019). However, the current found little evidence that athletes support each other when they migrate to online training environments. The lack of physical contact with other athletes meant that social cohesion in online classes was difficult to achieve. Online classes provided some athletes with a means of maintaining their physical fitness but they did little to foster social relationships.

Past research has raised fears that athletes may experience a loss in motivation during lockdown (Clemente-Suárez et al., 2020; Iancheva et al., 2020; Samuel et al., 2020). Indeed, research confirms that lockdown conditions result in decreased intrinsic goal striving among high level athletes (Martínez-González et al., 2021). Such decreases in motivation put high level athletes at risk of developing adjustment disorder. Therefore, adjustment to lockdown conditions seems to depend on mobilisation of individual resources that help to mitigate against the situational stressors. Resilience may be a key factor in this regard. Interestingly, research on amateur athletes has found that the vast majority retained moderate-to-high levels of commitment to their sports during lockdown (Angosto et al., 2020). Our results suggest that experienced endurance athletes [ranging from competitive amateurs to (semi)-professional athletes] also remained committed to their sports during lockdown. We had hypothesised that athletes with high athletic levels (personal commitment to endurance sports) would exhibit greater lockdown resilience and general coping abilities (H2a), as well as lower perceived barriers to training (H2b). Analyses revealed that endurance athletes who displayed greater athletic levels were indeed more likely to exhibit greater lockdown resilience and adaptive cognitive-emotional adaptive, coping strategies. These athletes also perceived lower barriers to training during lockdown. Netherthless, it is clear that the lockdown hardship experienced by amateur and elite athletes is different. Inactivity and the risk of adjustment difficulties are likely to be much higher for elite athletes who have more to lose from disrupted training plans. The present study used a measure of resilience (RISC-25) which focused on an individual’s ability to regain biological and psychological equilibrium (Connor and Davidson, 2003) by maintaining goal focus in challenging circumstances. In our study, athletes who exhibited high resilience levels also perceived lower barriers to exercising during lockdown. It is clear that the contextual adversity faced by our sample of mainly amateur athletes is different to that faced by our smaller sample of high level, competitive athletes. Nevertheless, resilience appears to be central to helping athletes from various sporting backgrounds and abilities in adjusting to adversity.

### Limitations

The current study has a number of limitations. First, our study did not analyse the psychological characteristics of the general population. This comparison between the general public and athletes might have proved insightful as it would have helped to determine how resilience levels and coping strategies vary between cohorts exposed to a pandemic level event. Second, the methodological tool used to measure resilience (i.e., RISC-25) potentially poses problems as it is not specifically adapted to sport. However, it should be noted that this tool has already been widely used to study resilience in athletes. Third, the impact on training routine was assessed in a quantitative manner (i.e., volume contrast) and questions still remain with regard to the specific reasons behind changes in training volume. A qualitative approach to investigating of these changes to training routine might provide further insights into athlete coping during lockdown. Fourth, in our study, the relative strictness of mobility restrictions in different countries was not based on objective criteria. Instead, strictness was only assessed through individual subjective appraisals. Therefore, a negative perception could not only be due to lockdown restrictions but could also be influenced by personality traits. For example, individuals exhibiting high levels of neuroticism would likely perceive mobility restrictions more negatively than those exhibiting low levels of neuroticism. In our defence, the aim of the current study was to purposefully focus on athletes’ subjective experiences of lockdown. Fifth, individual characteristics such as personality traits, past mental illness or physical disability were not measured in the current study. Our study focused on athletes’ current adaptive capacity within the context of COVID-19 restrictions. Specifically, the CERQ was used to assess the adaptive and maladaptive cognitive-emotional strategies that participants used when dealing with the lockdown. Finally, the stressors considered in the present study concerned mainly those that disrupted training regimes and athlete’s subjective perceptions of mobility restrictions. Our narrow focus on these stressors precluded the inclusion of other potentially relevant lockdown stressors. For example, Simons et al. (2021) identified a number of lockdown stressors among a small sample of elite athletes in Australia; relocation, self-quarantine and social distancing, uncertainty about the future, decreased income, changed university teaching methods, training facilities being unavailable, or season/competition being cancelled. Obvious omissions notwithstanding, most athletes in the current study did not compete at a high level, so the case for including stressors pertaining to competition and commercial success seems questionable.

### Practical Implications

The current research has practical implications for endurance athletes. In the words of Shakespeare, “there is nothing either good or bad, but thinking makes it so.” Athletes who can learn to reframe the mobility restrictions as an opportunity to rest, recover, and find new avenues for personal growth will likely be able to weather the “slings and arrows of outrageous fortune.” The disruption caused by the COVID-19 pandemic may be viewed as an opportunity to rethink and restructure training habits that have gone unchallenged or unquestioned. It is worth noting that it is the unthinking nature of our everyday lives that permits poor habits to persist. Past research shows that dramatic changes in lifestyle are catalysts for habit change (Wood et al., 2005). Therefore, when daily routines are disrupted, old and counter-productive (training) routines can potentially be replaced by new and improved ones. Athletes who are aware that much of their daily lives are habitual in nature (Wood et al., 2002) will be better placed to enact positive changes to disrupt the unthinking, status quo. A global pandemic which alters the very fabric of everyday life is likely to provide fertile conditions for behaviour change. In such situations, old habits can be broken and new and improved habits can be installed to accommodate the “new normal.”

Research suggests that while moderate-to-vigorous PA levels tend to rebound post-lockdown, their engaging in low levels of PA do not tend to maintain these modest levels of exercise after lockdown (Di Sebastiano et al., 2020). Furthermore, past research has found that active people tend to increase their PA levels as a coping response to stress (Stults-Kolehmainen and Sinha, 2014). Some of the reasons for the divergence in coping strategies may be explained by habitual behaviours. Habitual behaviours (e.g., exercise routines) can often be very robust (Dean, 2013). Therefore, an individual’s propensity for engaging in exercise might possibly be linked to their pre-pandemic exercise routines and habits. This may be partly due to the fact that ingrained habits and routines are psychologically comfortable (Avni-Babad, 2011). Research suggests that athletes who are intrinsically motivated to exercise are more likely to continue exercising during lockdown (Leyton-Román et al., 2021). Since endurance athletes spend long hours training, they are already likely to be intrinsically motivated to exercise. This assertion supports the findings of Lesser and Nienhuis (2020) who found that lockdown conditions tend to reinforce pre-existing behavioural patterns. If this is the case, then it is amateur athletes and those who do not exercise regularly who would benefit most from a lockdown induced reassessment of their habits. Nevertheless, all athletes, regardless of proficiency, would do well to view disruptive life events as opportunities to question, reconsider, and reformulate their approaches to training.

### Conclusion

In this study, we explored factors that influenced how a sample of mainly amateur endurance athletes perceived barriers to training during COVID-19 lockdown. Since endurance athletes engage in high levels of PA within outdoor environments, we postulated that lockdown conditions would be especially difficult for this cohort. We also hypothesised that athletes’ lockdown resilience and athletes’ general coping strategies (i.e., emotion regulation strategies) would influence their subjective perceived barriers to training. The current research demonstrates that endurance athletes perceive elevated barriers to training if: (1) they exhibit low lockdown resilience (H1a), (2) if they experience a negative impact of lockdown restrictions on their training hours (H1c), and (3) if they hold negative perceptions about lockdown mobility restrictions (perceived strictness; H1d). The present study also confirmed other hypotheses. Specifically, athletes with a high personal commitment to endurance sports exhibited greater lockdown-related resilience, greater coping abilities (H2a), and also perceived lower barriers to training (H2b). These findings suggest that resilience is critical to promoting functional adaptation to aversive lockdown conditions. The results also suggest that individual dispositions which regulate emotions also have a role to play in shaping perceptions of adversity. Therefore, levels of event-specific resilience and general emotion regulation appear to interact when overcoming adversity. Our findings suggest that the more committed endurance athletes are, the more likely they are to prevail when faced with challenges that jeopardise the pursuit of their sporting activity. Future research should aim to understand the specific contributing variables to this phenomenon. For example, future research could investigate internal factors (e.g., personality traits and self-efficacy) and external factors (e.g., the nature of the athlete’s commitment and their ability to access training facilities) that might influence their responses. Such considerations are important since they are likely to vary between different levels of athlete. For example, barriers to training are likely to assume an additional weight if they severely compromise the athlete’s ability to earn a livelihood. Accordingly, it seems entirely feasible that the inclusion of more elite athletes in our study might have revealed a reverse relationship between athletic levels and perceived barriers to training. While the present study provides a valuable cross-sectional snapshot in time, we nevertheless acknowledge that a longitudinal investigation might have revealed a different interplay between variables. One is left to wonder about the specific effects of lockdown severity on athlete coping strategies and a host of other intriguing questions. Thankfully, at the time of writing, these questions matter not. However, pandemics and lockdowns are likely to become more commonplace. Future research might profitably explore the linkages and interdependencies between other psychological constructs and different situational variables. Such research could help athletes to cope with future lockdowns. While the spectre of lockdown has receded in recent months, it nevertheless seems prudent to prepare for the unpalatable proposition of lockdowns yet to come.

## Data Availability Statement

The original contributions presented in the study are included in the article/supplementary material; further inquiries can be directed to the corresponding author.

## Ethics Statement

This study involving human participants was reviewed and approved by the Faculty of Business and Law at De Montfort University. The participants provided their written informed consent to participate in this study.

## Author Contributions

BH, GD, LP, and RAP contributed to the conception and design of the study and co-wrote the manuscript. GD organised the database and performed the statistical analysis. All authors contributed to the article and approved the submitted version.

## Funding

Open access funding was provided by the University of Lausanne.

## Conflict of Interest

The authors declare that the research was conducted in the absence of any commercial or financial relationships that could be construed as a potential conflict of interest.

## Publisher’s Note

All claims expressed in this article are solely those of the authors and do not necessarily represent those of their affiliated organizations, or those of the publisher, the editors and the reviewers. Any product that may be evaluated in this article, or claim that may be made by its manufacturer, is not guaranteed or endorsed by the publisher.

## Nomenclature

Resource Identification Initiative
PA: Physical activity
ITRA: International trail running association
WHO: World Health Organisation.
